# Onco-hypertension: hypertension induced by VEGF pathway inhibition

**DOI:** 10.3389/fcvm.2026.1748445

**Published:** 2026-04-28

**Authors:** Nicolas L. Palaskas, Jung Hyun Kim, Silvia Fernanda López Moreno, Bernardo Casso-Chapa, Noah I. Beinart, Nnenne Nnanna, Keila Carolina Ostos Mendoza, Anita Deswal, Syed Wamique Yusuf, Efstratios Koutroumpakis, Jun-ichi Abe, Michael S. Ewer

**Affiliations:** Department of Cardiology, The University of Texas MD Anderson Cancer Center, Houston, TX, United States

**Keywords:** blood pressure, cancer, cardio-oncology, hypertension, vascular endothelial growth factor

## Abstract

Cancer therapies are increasingly linked to a wide spectrum of cardiovascular toxicities, presenting significant challenges in the long-term care of oncology patients. Among these, therapy-induced hypertension stands out as one of the most consistent and clinically relevant adverse effects, especially with agents targeting the VEGF pathway. Understanding the mechanisms behind this form of hypertension is critical, as it not only represents a modifiable cardiovascular risk factor but may also serve as a biomarker of therapeutic efficacy in certain cancer treatments. Clinical evidence underscores the importance of early detection and aggressive blood pressure control to improve both cardiovascular and oncologic outcomes. The most well-characterized and clinically significant subtype of therapy-related hypertension is that induced by VEGF pathway inhibitors, owing to its high incidence, rapid onset, and well-defined mechanistic basis. In this review, we specifically examine VEGFi-associated hypertension as a prototypical model of onco-hypertension. Evidence-based strategies for managing therapy-induced hypertension emphasize early detection and individualized treatment plans. Renin-angiotensin system inhibitors (RAAS inhibitors) are frequently recommended as first-line agents due to their favorable cardiovascular profile and potential synergistic effects with some cancer therapies. Calcium-channel blockers (CCBs) also demonstrate strong efficacy and are often used in combination regimens to achieve optimal blood pressure control. Successful management requires a multidisciplinary approach, integrating expertise from oncology, cardiology, and primary care. Proactive surveillance, patient education, and risk stratification are essential components of care. As the field of cardio-oncology continues to evolve, structured blood pressure management remains a cornerstone of safe and effective cancer care, ensuring that patients receive optimal therapeutic benefit while minimizing cardiovascular risk.

## 1 Introduction

Cancer therapeutics encompass a broad spectrum of cardiovascular toxicities, presenting significant challenges in the longitudinal care of oncology patients. As highlighted in our core curriculum lecture series, specific agents such as HER2-directed therapies and anthracyclines are well known to induce cardiomyopathy. Additionally, thoracic radiation therapy has been associated with late-onset valvular heart disease and coronary artery disease—complications that may manifest years after initial exposure and require vigilant long-term surveillance.

Within this complex landscape of cardio-oncology, hypertension has emerged as one of the most consistent and clinically significant adverse effects. Cancer therapy related hypertension (CTRH) can result from multiple drug classes, including corticosteroids, calcineurin inhibitors, alkylating agents, anthracyclines, proteasome inhibitors, and radiation-associated vascular injury. The most well characterized and clinically significant CTRH subtype is that induced by vascular endothelial growth factor (VEGF) pathway inhibitors. The link between VEGF inhibition and hypertension is well established and has been emphasized in both clinical practice and cardio-oncology guidelines. Hypertension serves not only as a biomarker of VEGF pathway blockade but also as a toxicity that necessitates structured monitoring and management to mitigate long-term cardiovascular risk (1–4).

The purpose of this review is to provide a focused and comprehensive examination of VEGF pathway inhibitors, focusing on their epidemiologic impact, the pathophysiology of treatment-induced hypertension, strategies for surveillance, and evidence-based approaches to management. This discussion aims to equip clinicians with the knowledge necessary to recognize, monitor, and address VEGF-related hypertension, thereby improving cardiovascular outcomes in patients undergoing cancer therapy.

## 2 Basic pathophysiology of VEGF

**Figure 1:**
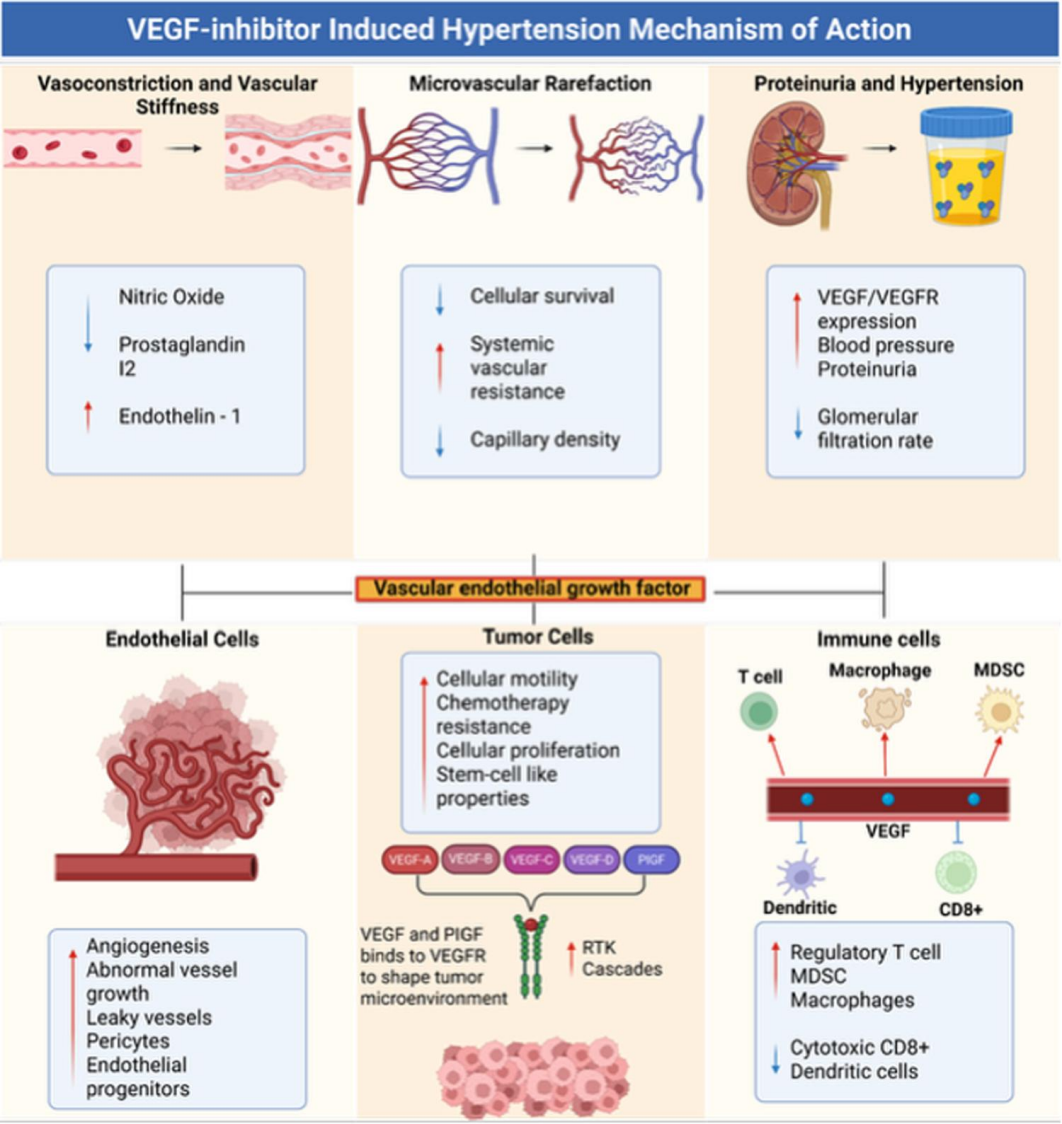
Potential mechanisms of VEGF inhibitor-induced hypertension.

The pathophysiologic rationale for targeting the VEGF pathway is first reviewed, with emphasis on its central role in tumor angiogenesis. Tumor vessels are structurally and functionally abnormal, with disrupted endothelial junctions, pericyte loss, increased permeability, and a disorganized architecture. These aberrations sustain tumor growth and impede drug delivery. Anti-VEGF therapies, pioneered through the discoveries of Ferrara and colleagues, are built in this conceptual framework and have transformed the therapeutic landscape of multiple malignancies (5, 6). Yet, the same VEGF-dependent pathways are critical for vascular homeostasis. Their pharmacologic inhibition reduces nitric oxide bioavailability, increases vascular stiffness, and induces microvascular rarefaction, all of which are proposed mechanisms for producing new-onset or worsening hypertension (7). The first mechanism described involves a decrease in nitric oxide and prostaglandin I2 and an increase in endothelin-1, all of which led to increased vasoconstriction (7). (REF) A decrease in capillary beds by VEGF therapy, also known as capillary rarefaction, is also described as a possible mechanism by increasing systemic resistance (7, 8). (REF) Lastly, VEGF therapy also possibly affects kidney podocytes and mesangial and endothelial cells, decreasing glomerular filtration rate and damaging renal vasculature, all which raise blood pressure (9) (Figure 1).

Proteinuria, which can be defined as >2+ protein on dipstick or urinary protein-to-creatinine ratio (UPCR) of ≥1 g/g, is a well-recognized toxicity of VEGF pathway inhibition. Physiologically, proteinuria reflects structural and functional injury to the glomerular filtration barrier through podocyte effacement, endothelial damage, or loss of slit diaphragm integrity resulting from VEGF blockade. These renal changes activate the renin-angiotensin-aldosterone system (RAAS), increase sympathetic nervous system activity, and reduce nitric oxide bioavailability. Collectively, these mechanisms enhance systemic vasoconstriction, sodium and water retention, and vascular stiffness, ultimately contributing to treatment-related elevations in blood pressure (10).

The increasing use of immune checkpoint inhibitors (ICI) and VEGFi combination therapies has raised questions regarding potential additive toxicity. Current evidence indicates that hypertension in these regimens remains predominantly driven by VEGFi. A meta-analysis of six phase III RCTs (*10.3389/fonc.2021.739263*) found no significant increase in all-grade or high-grade hypertension with ICI+ VEGFi combinations compared with VEGFi monotherapy. Pharmacovigilance data from FAERS (*10.3389/fimmu.2023.1127128*) similarly demonstrated a strong hypertension signal for VEGF inhibitors and for ICI+ VEGFi combinations, whereas ICIs alone showed no hypertension signal (IC025 −1.456; ROR025 0.368). Mechanistically, ICIs may potentially increase the risk of hypertension indirectly, through immune-mediated nephritis. Conversely, VEGFi-induced hypertension may heighten susceptibility to cardiovascular immune-related adverse events, but synergistic hypertension has not been demonstrated clinically.

Beyond vascular growth, VEGF also shapes the immune microenvironment. By suppressing cytotoxic CD8+ T-cells, expanding regulatory T cells, and promoting myeloid-derived suppressor cells (MDSCs), VEGF facilitates tumor immune evasion (11, 12). This dual vascular and immunologic role has provided the conceptual framework for combining VEGF inhibition with immune checkpoint blockade, a strategy that has consistently demonstrated synergistic activity in preclinical studies and has translated into improved clinical outcomes across multiple tumor types (13, 14).

The VEGF signaling axis is heterogeneous, comprising multiple ligands (VEGF-A, -B, -C, -D, and placental growth factor) and receptors (VEGFR-1, -2, -3) as well as co-receptors such as neuropilins (15). This diversity contributes to context-dependent effects on both vascular and lymphatic biology and may partly explain variability in efficacy and toxicity across patients and agents. Importantly, VEGF also intersects with immune regulation. By suppressing cytotoxic CD8^+^ T cells, expanding regulatory T cells, and increasing myeloid-derived suppressor cells (MDSCs), VEGF promotes tumor immune evasion—mechanisms corroborated in translational studies and consistent with the synergy observed when VEGF blockade is combined with immune checkpoint inhibitors (16, 17) (Figure 1).

## 3 VEGF inhibitors and cancer

Clinically, two main classes of VEGF-pathway inhibitors are in widespread use. Monoclonal antibodies such as bevacizumab and ramucirumab that are administered intravenously, exhibit prolonged half-lives, and carry perioperative implications. For instance, bevacizumab is typically withheld for at least six weeks before major surgery to minimize impaired wound healing (Roche Medical Information, 2024). In contrast, small-molecule TKIs such as sunitinib, axitinib, cabozantinib, and lenvatinib are oral agents with rapid onset and offset of action. This pharmacokinetic profile not only influences efficacy but also shapes toxicity management, as blood pressure elevations may occur within hours of initiation and normalize quickly upon discontinuation (18).

Most TKIs are multitargeted agents. Lenvatinib, for example, inhibits VEGFR1-3 as well as FGFR1-4, PDGFR*α*, RET, and KIT; cabozantinib blocks VEGFR1-3, MET, AXL, and RET (18). Such broad kinase inhibition expands antitumor activity but also contributes to off-target vascular effects, reinforcing the importance of reviewing detailed drug information for anti-angiogenic activity whenever hypertension or vascular events arise. Even agents primarily developed against other targets, such as cobimetinib (a MEK inhibitor), may exhibit secondary VEGF-pathway activity sufficient to provoke hypertension (Table 1A).

**Table 1A:** Classes of VEGF inhibitors.

Class	Generation/Subcategory	Agents
Anti-VEGF Biologics	Systemic oncology	Bevacizumab, Ramucirumab, Aflibercept
	Ophthalmic VEGF inhibitors	Ranibizumab, Brolucizumab, Faricimab, Pegaptanib, Conbercept
VEGF-Targeted Small-Molecule TKIs/MKIs	First-generation multikinase inhibitors (broad targets; higher vascular toxicity)	Sorafenib, Sunitinib, Pazopanib, Vandetanib, Cabozantinib, Regorafenib, Nintedanib, Lenvatinib, Anlotinib
	Second-generation VEGFR-selective TKIs	Axitinib, Apatinib, Tivozanib
	Third-generation highly selective VEGFR inhibitors	Fruquintinib
Tyrosine Kinase Downstream Pathway Inhibitors	KRAS G12C inhibitors	Sotorasib, Adagrasib
	BRAF inhibitors	Vemurafenib, Dabrafenib, Encorafenib
	MEK inhibitors	Trametinib, Cobimetinib, Binimetinib, Selumetinib
	PI3K inhibitors	Idelalisib, Copanlisib, Duvelisib, Alpelisib
	mTOR inhibitors	Temsirolimus, Everolimus, Sirolimus

In current oncology practice, VEGF inhibitors are integral in renal cell carcinoma, hepatocellular carcinoma, thyroid cancer, and in advanced endometrial carcinoma, increasingly in combination with immune checkpoint blockade (17, 19). These regimens complicate attribution of adverse events; differentiating VEGF-induced hypertension with subsequent heart failure from ICI-associated myocarditis may be challenging. A growing number of newer TKIs, including tivozanib, have also entered clinical use; in pivotal trials, tivozanib demonstrated a high incidence of hypertension, with grade ≥3 events in approximately 20%–24% of patients, reinforcing the need for vigilance in later-line RCC therapy (20) (Table 1B).

**Table 1B:** Cardiovascular adverse events from VEGF inhibitors.

Drug	Side Effect(s)						Frequency
**Monoclonal Antibodies**	**HTN**	**HF**	**↑QTc**	**VTE**	**ATE**	**MI**	
Aflibercept	**X**				**X**	**X**	**X** → ≥10%, very common
Bevacizumab	**X**	**X**		**X**	**X**	**X**	
Ramucirumab	**X**				**X**	**X**	
**VEGF TKI**							
Axitinib	**X**	**X**		**X**	**X**	**X**	**X** → 1 to <10%, common
Cabozantinib	**X**	**X**	**X**	**X**	**X**	**X**	
Lenvatinib	**X**	**X**	**X**		**X**	**X**	
Pazopanib	**X**	**X**	**X**	**X**	**X**	**X**	**X** → 0.1 to <1%, uncommon
Regorafenib	**X**	**X**			**X**	**X**	
Sorafenib	**X**	**X**	**X**			**X**	
Sunitinib	**X**	**X**	**X**	**X**		**X**	**X** → <0.1%, rare
Vandetanib	**X**	**X**	**X**			**X**	

## 4 VEGF-TKI-induced hypertension

Across this therapeutic class, hypertension remains the most common cardiovascular toxicity, affecting 10%–60% of treated patients (3). While the 2022 ESC Cardio-Oncology Guidelines describe hypertension as the signature toxicity of VEGF inhibition, other clinically relevant events include QTc prolongation (particularly with vandetanib), venous thromboembolism (notably with bevacizumab), arterial thromboembolic complications, myocardial infarction, and, in some cases, direct cardiomyopathy independent of blood pressure elevation (3, 4). Observational data suggest that arterial occlusive risk is particularly pronounced with non-VEGF TKIs such as nilotinib and ponatinib, while imatinib remains relatively vascular sparing (21).

The lecture also emphasized the rapid kinetics of VEGF-TKI-induced hypertension: cases of blood pressure exceeding 180 mmHg may occur within the first day of lenvatinib initiation, with reversal upon treatment interruption (22). This dynamic requires careful titration of antihypertensive therapy to prevent symptomatic hypotension during treatment holds, such as those prompted by gastrointestinal toxicity. A clear dose–response relationship is also observed; oncologists frequently reduce cabozantinib from 80 mg to 40 mg daily when blood pressure remains uncontrolled (23).

From a cardio-oncology standpoint, however, the emphasis should be on rapidly optimizing antihypertensive regimens rather than compromising oncologic efficacy through premature dose reductions (3, 24). Institutional series further refine this perspective. In a Stanford cohort with more than 3,000 longitudinal blood pressure measurements, axitinib was associated with the largest increases in both systolic and diastolic values, followed by sunitinib (9). Calcium-channel blockers and potassium-sparing diuretics proved particularly effective in mitigating these effects, highlighting the importance of proactive, evidence-based management strategies.

The highest predictors of who will develop VEGF-induced hypertension include those with pre-existing hypertension. Advanced age, possibly linked with already damaged capillary beds and vascular resistance, is also an important predictor. The use of multi-kinase inhibitors, also increases the likelihood of drug-induced hypertension, most probably due to their targeting of multiple VEGF receptors. Other factors are mentioned, such as kidney disease or genitourinary cancer, but are likely linked to other issues (10).

## 5 Definition of hypertension

The definition of hypertension is given according to the Common Terminology Criteria for Adverse Events (CTCAE) criteria, which ranges from grade 1 to grade 5, where grade 1 includes all adults with systolic blood pressure (SBP) ranging from 120 to 139 mmHg and DBP ranging from 80 to 89), while grade 5 indicated patient death (25). Treatment by a cardiologist is usually reserved for when a patient reaches grade 3 (Table 2A). This classification differs from the one proposed by the American College of Cardiology/American Heart Association (ACC/AHA) guidelines, where it is graded as normal, elevated, or stage 1 or 2 hypertension (26]), all with lower thresholds when compared to CTCAE criteria (Table 2B). These lowers thresholds are derived from the SPRINT trial, as described later.

**Table 2A:** Hypertension Grading Scale CTCAE.

Grade	BP (mmHg)
1	–
2	Systolic BP 140–159
	mmHg or diastolic BP 90–99
	mmHg; recurrent or persistent
3	Systolic BP 160–179 mmHg or diastolic BP 100–109 mmHg persisting over 1 h; SBP >140 and plus either increase in SBP >20 mmHg or increase MAP >15 mmHg from baseline
4	SBP≥180 or DBP ≥110 mmHg persisting over 1 h; BP associated with acute hypertension mediated organ damage; lifethreatening consequences (e.g., malignant hypertension, transient or permanent neurologic deficit, hypertensive crisis); urgent intervention indicated
5	Death

**Table 2B:** Hypertension Grading Scale AHA/ACC.

Grade	Systolic BP (mmHg)	|	Diastolic BP (mmHg)
Normal	<120	AND	<80
Elevated	120–129	AND	<80
HTN Stage 1	130–139	OR	80–89
HTN Stage 2	≥140	OR	≥90

*Patient with systolic or diastolic BP in HTN stage 2 → designate higher grade*.

The SPRINT trial, composed of 9,361 patients randomized into intensive and standard blood pressure control with the primary outcome being a composite of severe cardiovascular events. While both groups were able to reach their blood pressure goals, the intensive control group required 2–3 antihypertensive medications compared to 1–2 in the standard group, which really impacted the cardiovascular outcomes (Hazard ratio of 0.75 for a decreased composite of CVD and hazard ratio of 0.73 of mortality by any cause with intensive treatment), leading to a change of guidelines that favors a more intensive treatment of blood pressure (27). While the SPRINT trial evaluated the benefits of blood pressure control in the non-diabetic general population of patients ≥ 50 years of age, the conclusions are likely to be similar in the cancer population**.**

The European Society of Cardiology (ESC) Cardio-Oncology Guidelines (4) state baseline assessment recommendations for patients initiating VEGF inhibitor therapy. The initial step is to systematically stratify patients based on comorbidities as low, moderate, high or very high cardiovascular risk. A baseline electrocardiogram (ECG) reading is strongly recommended for all patients, regardless of risk, while a transthoracic echocardiogram (TTE) is only encouraged in high-risk patients (Class I recommendations). TTE may be considered in lower risk groups but is not required (Class IIa recommendation). In general clinical practice, all patients will get a TTE if they do not have one within a year of starting VEGF therapy. The ESC guidelines recommend getting natriuretic peptides such as BNP or NT-proBNP for patients at moderate to very high risk for the early detection of heart failure. Natriuretic peptide levels, however, do not always correlate with filling pressures or heart failure and have limited predictive value for VEGF inhibitor therapy related heart dysfunction and therefore are weakly recommended (Class IIb—IIa recommendation). Regardless, patients undergoing VEGF inhibitor therapy are at constant risk of VEGF inhibitor therapy induced cardiac dysfunction. Therefore, baseline cardiovascular assessment remains crucial for these patients**.**

Importantly, both the ESC and our baseline clinical visit guidelines strongly underscore the use of home blood pressure monitoring logs and patient education. The lecture emphasizes that patients need to be instructed on proper blood pressure measuring techniques. Additionally, our clinical practice recommends patients take two blood pressure measurements per day per day and to properly log the readings for trend recognition without focusing on any single high or low reading. Furthermore, all patients on VEGF inhibitor therapy should be taught about symptom-driven assessment of medical emergencies such as recognizing signs and symptoms of stroke and heart failure to prevent any negative cardiovascular events and timely attend medical emergencies. Lastly, our baseline clinical visit guidelines recommend reviewing the cardiovascular risk of every patient including lipid levels and baseline control of blood pressure before initiating VEGF inhibitor therapy.

## 6 How to treat TKI-induced hypertension?

One of the primary management goals for patients receiving VEGF inhibitors is to make sure you keep the patient on the VEGF inhibitor preferably maximum tolerated dose (limit dose reductions). This is because dose reductions or interruptions can compromise cancer outcomes (28). Our approach is to keep the blood pressure at or less than 140 mmHg consistent with the European Society of Cardiology guidelines (4).

According to NCI clinical trial protocols, patients starting VEGF inhibitors should have blood pressure monitoring during the first cycle, followed by every 2–3 weeks while on therapy (24). This is because hypertension is a well-documented effect of the VEGF blockade and a major toxicity of the treatment (24).

The ESC guidelines further stratify blood pressure management based on cancer prognosis (4). The ESC outlines different blood pressure management if the patient is cancer free, curable cancer during treatment, and various metastatic cancer prognosis depending on the time of metastasis. It is also important to have an individualized approach that balances oncologic efficacy with cardiovascular safety (4).

**Figure 2:**
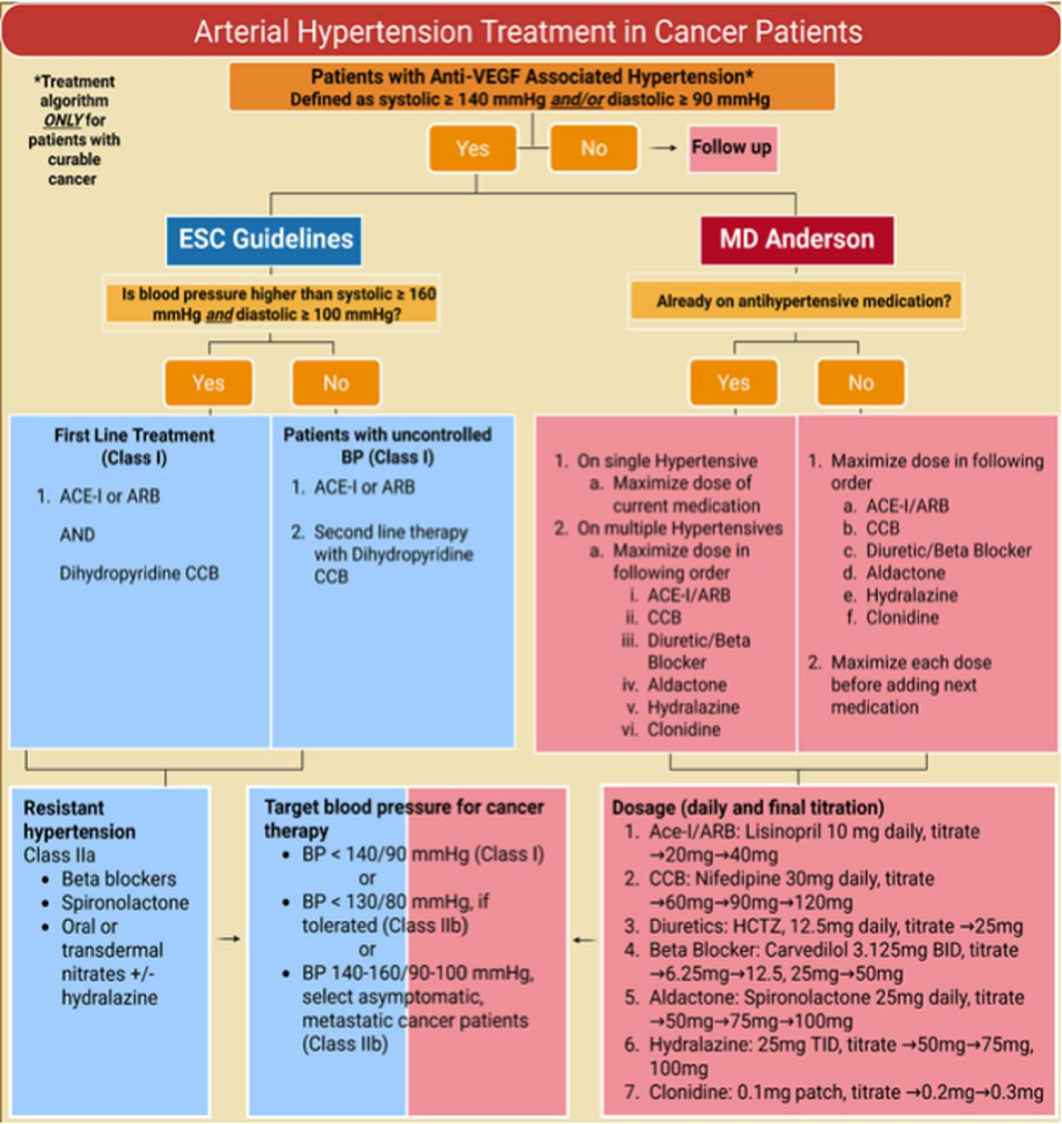
Treatment algorithms for VEGF Inhibitor-induced hypertension.

The choice of antihypertensive medications in VEGF inhibitor-induced hypertension is critical (4). The ESC has an algorithm to treat hypertension based on the systolic and diastolic blood pressure. The algorithm suggests starting with both as angiotensin-converting enzyme inhibitor (ACE-I) and a calcium channel blocker (CCB), but our approach typically avoids starting with both agents at once, aiming instead to maximize the dose of one drug before adding another. Our preference is often given to beginning with renin-angiotensin-aldosterone system (RAAS) inhibitors followed by CCBs, though the literature remains mixed on which medication should be used first (4, 29). If still resistant to hypertension, the ESC recommends giving non-selective beta-blockers can be used, then spironolactone, and then nitrates and/or hydralazine (4) (Figure 2).

According to the ESC Cardio-oncology Guidelines, the preferred sequence for initiating antihypertensive therapy in patients undergoing cancer treatment begins with RAAS inhibitors**,** such as ACE inhibitors (ACE-Is) or angiotensin receptor blockers (ARBs), followed by calcium channel blockers (CCBs)—specifically amlodipine or nifedipine. For patients presenting with systolic blood pressure ≥160 mmHg and diastolic blood pressure ≥90 mmHg, combination therapy using both a RAAS inhibitor and a dihydropyridine CCB is recommended to achieve optimal blood pressure control. The importance of understanding the pathophysiological mechanisms underlying treatment-related hypertension cannot be overstated. For example, certain TKIs are known to reduce nitric oxide production, which contributes to endothelial dysfunction and proteinuria. This effect is particularly concerning in patients with advanced chronic kidney disease (CKD), and clinicians are advised to proceed cautiously in this population (10).

RAAS inhibitors play a dual role by not only lowering blood pressure but also mitigating proteinuria and preserving renal function. Dr. Palaskas emphasizes the importance of monitoring renal parameters when initiating RAAS therapy, especially in patients with normal baseline kidney function. A transient increase in serum creatinine (up to 30%) is expected and typically stabilizes over time. This physiological response should not be misinterpreted as acute kidney injury; the patient be closely monitored, and the rise in creatinine should remain within an acceptable range (10).

In a retrospective study of 213 metastatic renal cell carcinoma (mRCC) patients treated with first-line sunitinib at Gustave Roussy (2004–2013), hypertension was associated with longer overall and progression-free survival, while the use of angiotensin system inhibitor (ASI) independently conferred significant benefits in both outcomes. ASI use improved overall survival (HR = 0.40, *P* < 0.001) and progression-free survival (HR = 0.55, *P* = 0.009), with effects consistent regardless of treatment timing. These findings suggest concomitant ASI therapy may be an indicator of enhance survival in mRCC patients receiving sunitinib (30).

In a study of 4,736 mRCC patients, ASI use was linked to longer overall survival compared with other antihypertensives (26.7 vs. 18.1 months) and no therapy (26.7 vs. 16.7 months), with the greatest benefit seen in those receiving VEGF-targeted treatment. *In vitro*, sunitinib combined with an ASI significantly reduced RCC cell viability, supporting a potential therapeutic role for ASIs (31).

In a study of 228 mRCC patients treated with anti-VEGF TKIs, blood pressure increased significantly. Among antihypertensive agents, calcium-channel blockers (CCBs) and potassium-sparing diuretics produced the greatest BP reductions (9).

These reports suggested that ASIs and CCBs are effective in managing hypertension during VEGF-targeted therapy in mRCC patients. Beyond blood pressure control, ASIs have been consistently associated with improved overall and progression-free survival, suggesting a potential dual benefit in both cardiovascular and oncologic outcomes. ESC cardio-oncology guidelines emphasize that clinicians should anticipate the normalization of blood pressure after discontinuation of VEGF inhibitors and modify antihypertensive regimens accordingly, though they do not specify operational details. To operationalize this recommendation, we suggest applying a monitoring framework similar to that used during VEGFi initiation: daily home blood pressure measurements for approximately four weeks after treatment discontinuation. Patients should be instructed to temporarily hold antihypertensive medications if systolic blood pressure falls below 110 mmHg or diastolic below 60 mmHg continuously for one week, or immediately if systolic blood pressure falls below 90 mmHg. Antihypertensive agents should be tapered or discontinued in the reverse order in which they were initiated, prioritizing withdrawal of medications that were added specifically to control VEGFi-related hypertension. For patients with pre-existing hypertension, only the agents introduced during VEGFi therapy should be considered for tapering, while baseline chronic therapies are typically maintained. Review of the patient's home blood pressure log at follow-up can guide clinicians if further changes to pharmacologic management is warranted in accordance with hypertension management guidelines.

In patients receiving planned on-off VEGFi treatment cycles, antihypertensive medications may be held during off-therapy periods due to expected BP normalization. These patients should be counseled to resume daily blood pressure measurements at the start of their next treatment period, as a recurrent rise in blood pressure is expected with VEGFi re-initiation. This increase should prompt resumption of the antihypertensive medications previously required to control their VEGFi-related hypertension.

## 7 Cancer prognosis

Interestingly, the development of treatment-induced hypertension during VEGF inhibitor therapy has been consistently associated with improved clinical outcomes in patients with metastatic cancers. Evidence suggests that this hypertensive response may serve as a pharmacodynamic biomarker of effective VEGF pathway inhibition. Patients who developed hypertension while receiving VEGF-targeted agents demonstrated significantly longer progression-free and overall survival compared to those who did not. This correlation has been observed across multiple retrospective analyses, reinforcing the hypothesis that the pharmacologically-induced hypertension may reflects mechanisms that are associated with anti-angiogenic activity and therapeutic efficacy (32).

## 8 MD Anderson study algorithm

The MD Anderson Hypertension Management Algorithm for the sequential initiation or escalation of antihypertensives therapy is depicted in Figure 2. This algorithm may serve as a guide in patients undergoing VEGF inhibitor treatment. The algorithm stratifies patients based on their baseline antihypertensive use. For those not previously on antihypertensive medications, therapy is initiated using a preferred sequence with aggressive dose titration. Patients already on a single agent are advised to maximize the current dose before adding additional medications. For patients on multiple agents, dose escalation follows a structured order: beginning with RAAS inhibitors (ACEi/ARB), followed by calcium-channel blockers (CCBs), then diuretics or beta-blockers, and finally agents such as spironolactone, hydralazine, and clonidine. Consistent with current hypertension guidelines, ACE inhibitors and ARBs are considered equivalent first line agents for hypertension. While our algorithm shares the ESC cardio-oncology framework of initiating treatment at BP ≥140/90 mmHg and applying prognosis-based BP targets (Figure 2), it differs primarily in its use of a stepwise escalation strategy and its inclusion of explicit drug-specific dosing and titration pathways. The MD Anderson algorithm intentionally adopts a stepped-care approach for VEGFi-related hypertension to reflect the unique clinical context of oncology patients, in whom VEGFi regimens may involve on-off treatment cycles and may also change rapidly based on therapeutic response or toxicity. Escalating a single agent before adding additional medications helps limit polypharmacy in a population already receiving multiple concurrent treatments and facilitates a more controlled taper once VEGFi therapy is discontinued and blood pressure normalizes. When escalation beyond a single agent is required, patients are routinely transitioned to a single-pill combination to preserve adherence benefits. This pragmatic, oncology-specific rationale underpins the current algorithm, designed to optimize blood pressure control while accounting for disease severity, particularly in higher-risk cohorts within the prospective study population. A prospective cohort study to evaluate the clinical effectiveness of the MD Anderson Hypertension Management Algorithm in patients receiving VEGF inhibitor therapy is ongoing (NCT05108519). The study aims to assess blood pressure control and cardiovascular outcomes across different patient subgroups, based on their baseline antihypertensive status and treatment response.

It is important to note that the MD Anderson algorithm applies specifically to patients with curable malignancies, in whom achieving a blood pressure <140/90 mmHg remains the standard target. For patients receiving VEGFi in the context of metastatic or poor-prognosis disease, our institution individualizes blood pressure goals and accepts more permissive thresholds, <160/100 mmHg, consistent with contemporary cardio-oncology guidance. In these scenarios, the primary objective is to avoid symptomatic or severe hypertension while maintaining the ability to continue cancer therapy.

Progress in cardio-oncology research remains limited due to a scarcity of well-powered clinical studies. For example, the CARISMA trial (NCT04467021), a randomized study designed to compare standard vs. intensive blood pressure control (SBP <140 mmHg vs. <120 mmHg), has yet to enroll participants. Similarly, a prospective observational study investigating the cardiovascular effects of VEGF receptor inhibitors and immune checkpoint inhibitors (ICIs) was discontinued due to insufficient patient recruitment. These setbacks underscore the urgent need for robust clinical investigations to better understand the mechanisms of treatment-induced hypertension and to establish evidence-based management targets for patients receiving VEGF-targeted therapies.

## 9 Conclusion

VEGF inhibitors are staple treatments used in contemporary oncology, often in combination with other cancer therapeutics. However, VEGF inhibitor induced hypertension remains a significant and common clinical change that causes cardiotoxicity in patients. The information presented in this review supports the need for aggressive blood pressure control in these patients (target systolic <140 mmHg) for beneficial and optimized therapeutic outcomes. While most of the current evidence available underscores RAAS inhibitors as the first line treatment, offering both excellent blood pressure control and oncologic benefits, some studies favor the use of CCBs instead since CCBs provide the same benefits as RAAS inhibitors. However, regardless of priority given to either of these drugs, most patients experiencing hypertension related VEGF therapy ultimately require a combination therapy of both. Future studies, such as the clinical trials mentioned above are critical for the optimization of therapeutic strategies and understanding of VEGF inhibitor related hypertension. Nevertheless, until such studies are completed in greater detail and quantity, an early proactive blood pressure control and attentive cardiovascular monitoring are the essential elements for treating patients with VEGF inhibitor induced hypertension. Lastly, it must be emphasized that effective control of hypertension in patients receiving VEGF-targeted therapies has not been shown to compromise oncologic outcomes. On the contrary, maintaining optimal blood pressure is critical to reducing cardiovascular risk, minimizing treatment interruptions, and enhancing overall tolerability, while preserving the efficacy of anti-cancer therapy (4, 33, 34).
